# EZH2‐Mediated PHF10 Suppression Amplifies HMGB1/NF‐κB Axis That Confers Chemotherapy Resistance in Cholangiocarcinoma

**DOI:** 10.1111/jcmm.70363

**Published:** 2025-02-04

**Authors:** Qiushi Yin, Daning Lin, Weiqian Zeng, Shijing Gu, Chuangshi Zhu, Changfu Liang, Yan Yang

**Affiliations:** ^1^ Department of Hepatobiliary Pancreatic Surgery The First Affiliated Hospital of Hainan Medical University Haikou China; ^2^ Hainan Medical University Haikou China

## Abstract

Chemoresistance represents a major threat to the treatment of human cancers, including cholangiocarcinoma (CHOL). Aberrant epigenetic events contribute most to the progression of CHOL and chemotherapy efficacy. PHF10, one subunit of SWI/SNF complex, expressed highly in tumours that correlated with tumorigenesis. However, the roles of PHF10 in CHOL remains unclear. Here, we utilised the bioinformatic analysis to reveal that PHF10 expressed lowly in CHOL samples relative to normal tissues. Functionally, we demonstrated that PHF10 deficiency enhanced cell proliferation, migration and self‐renewal capacities of CHOL cells. PHF10 ablation further enhanced the chemoresistance of CHOL cells. The transcriptome analysis revealed that PHF10‐KO could notably alter several oncogenic crosstalk, including the NF‐kB signalling. As the top hit, HMGB1 mRNA expressions had the sharpest increase upon PHF10 deficiency. PHF10 coordinated with Setdb1 to mediate the H3K9me3 modifications on the HMGB1 promoter to suppress its expressions. Low PHF10 relied on HMGB1 to promote the progression of CHOL cells in vitro and in vivo. Furthermore, EZH2 mediated the H3K27me3 enrichment on the PHF10 promoter region that contributes to its low expressions. Lastly, the HMGB1 inhibitor (Glycyrrhizin) decreased proliferation rate of PHF10‐deleted cells in vitro and in vivo. Targeting HMGB1 rendered PHF10^low^ CHOL re‐sensitive to chemotherapy. Collectively, this study demonstrated that PHF10 functions as a tumour suppressor in CHOL, and is a novel target to predict and overcome chemoresistance.

## 1 Introduction

Cholangiocarcinoma (CHOL), also known as bile duct cancer, is a relatively rare but aggressive form of cancer that originates in the cells of the bile ducts [1]. In most cases, surgical resection remains the only curative option [2]. However, the majority of patients present at an advanced stage when not amenable to surgical resection [3]. Gemcitabine and cisplatin are most widely used chemotherapeutic agents for CHOL. However, due to the presence of drug resistance in the tumour, the median survival of patients receiving chemotherapy is only 12 months [4]. As a result, it makes sense to explore the molecular mechanisms of CHOL progression and metastasis and identify novel targets to overcome resistance for CHOL patients.

As is well documented, epigenetic alterations are considered to be highly correlated with cancer progression [5, 6]. As an essential histone methyltransferase that functions as an enzymatic subunit of the polycomb repressive complex, enhancer of zeste homologue 2 (EZH2) relied on different action modes to participate in multiple biological functions [7]. EZH2 mainly modulates expressions of downstream targets via trimethylation of histone H3 at lysine (K) 27 (H3K27me3) or as a transcription co‐factor [8, 9]. Recently, several inhibitors targeting EZH2 are investigated and under investigation for cancer therapy. Cheng Wang et al. have optimised the proteolytic targeting chimeras (PROTACs) precision targeting EZH2, named with U3i, to degrade PRC2 complex in triple‐negative breast cancer (TNBC) cells, which could notably induce apoptosis of breast cancer cells [10]. However, the roles of EZH2 in CHOL are largely unknown and urgently needed to be clarified.

As is well known, the specific mechanisms of SWI/SNF functions are not well completely investigated [11]. Core subunits such as BRG1 or BRM ATPase, BAF47, BAF155 and BAF170 participate in chromatin‐remodelling activities, while the roles of BAF57, BAF60, as well as PBAF specific subunits BAF200, BAF180, BAF45CD, or PHF10 (BAF45A) are mainly restricted to cell viability and development [12]. Subunits of PBAF or BAF have been demonstrated to exert roles in recruiting complexes to target genes via interacting with RNA polymerase complex, or transcriptional regulators [13]. The mammalian SWI/SNF complex could be involved in transcriptional regulations of a variety of genes based on the diverse subunit compositions. PHF10/BAF45a is a specific subunit of PBAF subfamily of SWI/SNF complexes and this non‐core subunit of PBAF is recruited to the complex at the final stage of its assembly [14]. PHF10 has the double C‐terminal PHD (DPF) domain and one similar domain has been found in other proteins. PHF10 could interact with acetylated residues in histone 3 (H3K14 and H3K9) and recruit these complexes to active chromatin [15]. As reported, PHF10 is demonstrated to be vital for maintenance of proliferation of mouse neuroblasts, haematopoietic precursors and transcriptional activation in myelogenesis [16]. In addition, Yongxiang Wang et al. have found that circ_0001023 promotes the progression of gastric cancer (GC) through modulating the miR‐409‐3p/PHF10 axis, suggesting that PHF10 is an oncogene in GC [17]. PHF10 could also interact with MYC and facilitate the recruitment of PBAF complex to target gene promoters, therefore, amplifying MYC transcriptional activation of genes involved in the cell cycle progression of melanoma cells [17]. However, high‐throughput sequencing data implicated that downregulation of PHF10 in uveal melanoma cell lines and tumours altered a number of biological pathways associated with development and adhesion [14]. These findings suggest a role for PHF10 as a novel tumour suppressor at Chr 6q27. As a result, PHF10 may play distinct functions in diverse malignancies. However, the roles of PHF10 in CHOL have not been reported and discovered. Whether Setdb1, a protein lysine methyltransferase and methylates histone H3 at lysine 9, interacts with PHF10 also needs to be further studied.

In this study, we demonstrated that PHF10 modulates CHOL progression and invasion via the HMGB1/p50 signalling. We also found that EZH2 mediates the epigenetic suppression of PHF10 in CHOL cells via H3K27me3 modifications. Our study thus implicated that PHF10 might be a tumour suppressor in CHOL proliferation and metastasis, highlighting a potential therapeutical target for CHOL treatment.

## 2 Methods and Materials

### 2.1 Cell Lines and Culture

CHOL cell lines (HuH28, HuCCT1, RBE, HIBEC, TFK, QBC939, KMCH‐1) and 293 T cells were matained in DMEM medium. The medium was supplemented with 10% fetal bovine serum (FBS) and antibiotics (penicillin and streptomycin). These cells were maintained in medium at 37°C in a humidified atmosphere of 5% CO_2_.

### 2.2 Collection of CHOL Patient Samples

The CHOL samples and matched normal adjacent tissues were obtained from the Department of Hepatobiliary Pancreatic Surgery, the First Affiliated Hospital of Hainan Medical University. Sample collection and usage were approved by the Ethics Committee of the First Affiliated Hospital of Hainan Medical University in accordance with the Declaration of Helsinki (Number: 2018(Keyan) No.2), and written informed consent was obtained from all enrolled patients. PHF10 expression was detected via immunohistochemical staining in 60 pateints.

### 2.3 Generation of Stable CRISPR/Cas9*‐*Mediated PHF10‐Knockout Cells

The plasmid pX459 was used to guide oligos targeting PHF10 gene. The HuCCT1 and RBE cells were plated and transfected with pX459 constructs overnight, then screened with 1 μg/mL puromycin for 3 days. The monoclonal cell line was isolated and obtained by seeding living cells in 96‐well plate. The knockout efficiency of PHF10 gene was determined via Sanger sequencing. The specific primers were listed as the following: sgPHF10#1: F: 5‐CACCGTTACGGAATACTCTTTATAA‐3′; R: 5’‐AAACTTATAAAGAGTATTCCGTAAC‐3′. sgPHF10#2: F: 5’‐CACCGTGAGCCCATTCGCCTCCTTT‐3′; R: 5’‐AAACAAAGGAGGCGAATGGGCTCAC‐3′.

### 2.4 CCK‐8, Cell Proliferation Assay and 3D Soft Agar Assay

Cell viability was tested by CCK‐8 assay following manufactory's manual. Cells were plated in 96‐well plates at the density of 2000 cells per well in 100 μL DMEM. After those treatment, CCK‐8 reagents were added to the culture medium for 12 h. Then the optical densities (OD450) of the wells were measured by a microplate reader at 450 nm. Colony formation assay in soft agar was carried out in a 6‐well plate. The base layer was made by mixing 1.2% agarose and equivalent volume of 2 × medium with 20% FBS. Then cells from each group were collected and suspended in medium containing 0.4% agarose and plated over the base layer in triplicate at a density of 1000 cells per well. 30 days later, the clones were observed apparently and the cells were iced for 4 h, and then stained by 0.02% crystal violet. Images were taken after staining.

### 2.5 Wound Healing Assay

The CHOL cells were seeded in each well of a 6‐well plate, and cells were scratched with a 1 mL pipette tip after confluent. After washed with PBS slightly, the images were captured by using a microscope equipped with a digital camera. The images were recorded again using the same microscope after 24 h.

### 2.6 Sphere Formation Assay

Cells were seeded on ultra‐low attachment 96‐well plates in spheroid medium supplemented with B27 supplements, 20 ng/mL EGF, 20 ng/mL bFGF, and 1% antibiotics. Cells grown under sphere‐forming conditions were treated with vehicle or the indicated compounds at varying doses. For using vehicle‐ or Evo‐treated cells, cells were grown under sphere‐forming conditions without drug treatment. Cells were incubated at 37°C and 5% CO_2_ for 2 weeks or until spheres formed and reached above 150 μm^2^. Spheres were imaged, and the diameter of spheres and the number of spheres above 30 or 100 μm in diameter were determined using ImageJ software.

### 2.7 Extraction of RNA and Quantitative RT‐PCR

Total RNA from cells was extracted from tissues using the TRIzol reagent (Invitrogen, USA). Then RNA was reverse transcribed into cDNA using the Superscript RT kit (TOYOBO, Japan). Primers were designed by Primer 3.0 software. qRT‐PCR was conducted using SYBR Green kit master Mix (TOYOBO, Japan). GAPDH was used as the internal reference gene.

### 2.8 Reporter Assay

CHOL cells were co‐transfected with HMGB1 firefly luciferase reporter plasmid, Renilla luciferase plasmid, PHF10 and Setdb1 expression plasmids by polyethylenimine (PEI) according to the manufacturer's instruction. After 24 h, cells were lysed and centrifugated, and luciferase activity was measured using the dual‐luciferase reporter assay system (Promega, Madison, USA). Relative HMGB1 promoter activity was calculated as firefly luminescence relative to Renilla.

### 2.9 Western Blot

Western blot analysis was performed to examine the protein expression level. CHOL cells were homogenised with RIPA lysis buffer containing a complete protease inhibitor mixture. Cell lysates or immunoprecipitates were subjected to SDS‐PAGE, and then proteins were transferred onto nitrocellulose membranes (GE Healthcare). The membranes were blocked in 5% nonfat milk for 1 h and then incubated with primary antibodies specific for 12 h at 4°C overnight. After adequate washing with 1% TBST for 30 min, the membranes were incubated with secondary antibodies for 1 h. Finally, they were washed with 1% TBST and detected by a chemiluminescence system.

### 2.10 Immunohistochemistry (IHC)

The tissue slides were washed three times with PBS and fixed with 4% paraformaldehyde for 15 min and then permeabilized with 0.2% Triton X‐100 for 10 min at room temperature. Normal goat serum was added to the slides and incubated for 30 min at room temperature. After blocking with blocking solution for 1 h at room temperature, slides were incubated in diluted primary antibody overnight at 4 °C. The next morning, after incubation with biotinylated goat anti‐rabbit or anti‐mouse IgG at room temperature for 30 min, immunostaining was visualised with diaminobenzidine, and sections were then counterstained with haematoxylin.

### 2.11 Chromatin Immunoprecipitation

Cells (4 × 10^6^) in a 10 cm culture dish were treated with 1% formaldehyde to cross‐link proteins to DNA, and the reaction was stopped by glycine. The cell lysates were sonicated to shear the DNA to fragments of 300–1000 bp. Chromatin supernatants were incubated with anti‐Setdb1, anti‐H3K9me3 or anti‐immunoglobulin G antibody overnight at 4°C with rotation. After reversing the cross‐linking of protein/DNA complexes to free DNA, enrichment was analysed by qRT‐PCR.

### 2.12 RNA‐Seq

First‐strand cDNA was synthesised using reverse transcriptase and random primers, and residual RNA was synthesised using RNA. Then, DNA polymerase and dNTPs were used to synthesise the second‐strand cDNA. The 3′ end of the synthesised double‐stranded DNA fragment was adenosylated and subsequently connected to the splice hybridization site. Finally, PCR amplification was performed, and the product was purified with AMPure XP beads to obtain the library. Then Illumina NovaSeq 6000 was subsequently used for sequential analysis.

### 2.13 Bioinformatics Analysis

To identify differentially expressed genes, the R limma and edge packages were used for sequencing the differential expression of the genes in the matrix, for which |logFC| > 1 and the adjust *p* value < 0.05 were used. Then, GO and KEGG enrichment analysis of the differentially expressed genes was performed according to the clusterProfiler. Finally, ggplot2 and pheatmap were used for visualisation.

### 2.14 Animal Experiments

All animals in this study received humane care according to the National Institutes of Health guidelines. Animals were obtained from the SLAC Laboratory Animal Co. Ltd. All experimental protocols were approved in advance by the Committee for Animal Experimentation of the First Affiliated Hospital of Hainan Medical University (Nubmer: HYLL‐2020‐010).

### 2.15 Statistical Analysis

The data values were presented as the mean ± SEM. Differences in mean values between two groups were analysed by two‐tailed Student's t test. The *p* < 0.05 was considered to be statistically significant. The statistical analysis was conducted in Graphpad Prism (Version 8.0).

## 3 Results

### 3.1 PHF10 Expressed Lowly in CHOL That Predicts Poor Prognosis

**FIGURE 1:**
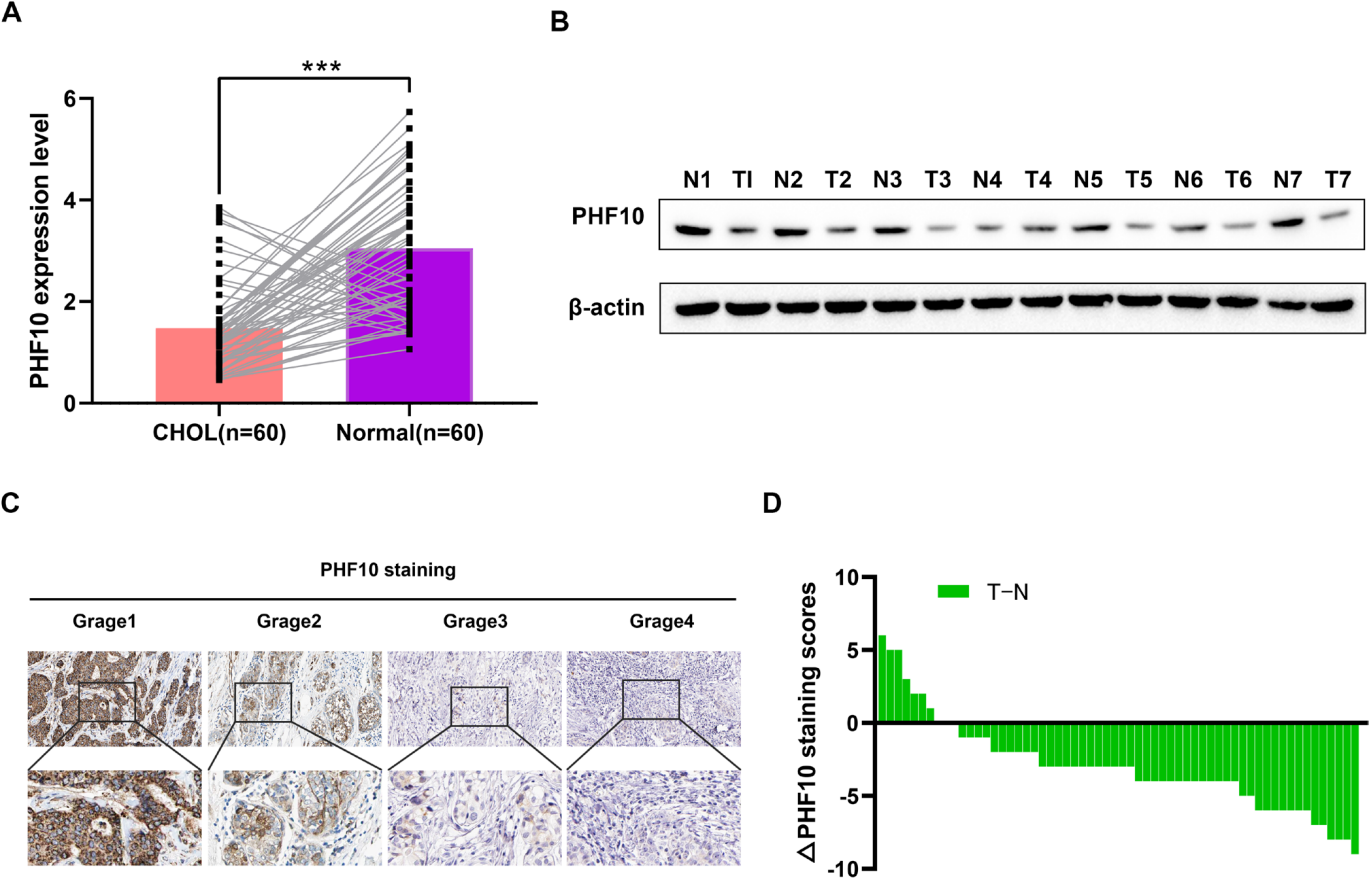
PHF10 was down‐regulated in CHOL that predicts poor prognosis. (A) Boxplot exhibiting the differential mRNA level of PHF10 in resected CHOL samples and matched tissues. (B) PHF10 protein level were detected in CHOL tissues and matched normal samples via western blotting (*n* = 7). (C) Representative IHC pictures on the CHOL samples with anti‐PHF10 antibody (scale bars = 200 μm or 50 μm, respectively) were exhibited. (D) The distribution of the difference in PHF10 H‐scores (△H‐score = T‐N). The △H‐scores of PHF10 staining was available in 60 pairs of tissues. *p < 0.05, **p < 0.01, ***p < 0.001.

To gain the clinical significance of PHF10 in CHOL, we collected a series of 60 CHOL samples with matched adjacent tissues from the First Affiliated Hospital of Hainan Medical University to verify that PHF10 expressions were notably lower in tumours compared with control samples (Figure 1A < 0.001). In addition, the protein levels of PHF10 were found to be notably lower in 6/7 (85.71%) human CHOL tissues than in their paired normal tissues based on results of western blot (Figure 1B). Low PHF10 expressions were also associated with advanced tumour grades of CHOL (Figure 1C and Figure S1). Immunohistochemical staining of 60 tissue pairs showed that the tumour tissue in most of the paired samples had a lower PHF10 score (Figure 1D). Taken together, our study suggested that PHF10 levels were down‐regulated in CHOL patients and low PHF10 might be an independent prognostic factor for patients with CHOL.

### 3.2 PHF10 Inhibition Promoted Cell Proliferation, Migration and Chemotherapy Resistance of CHOL

**FIGURE 2:**
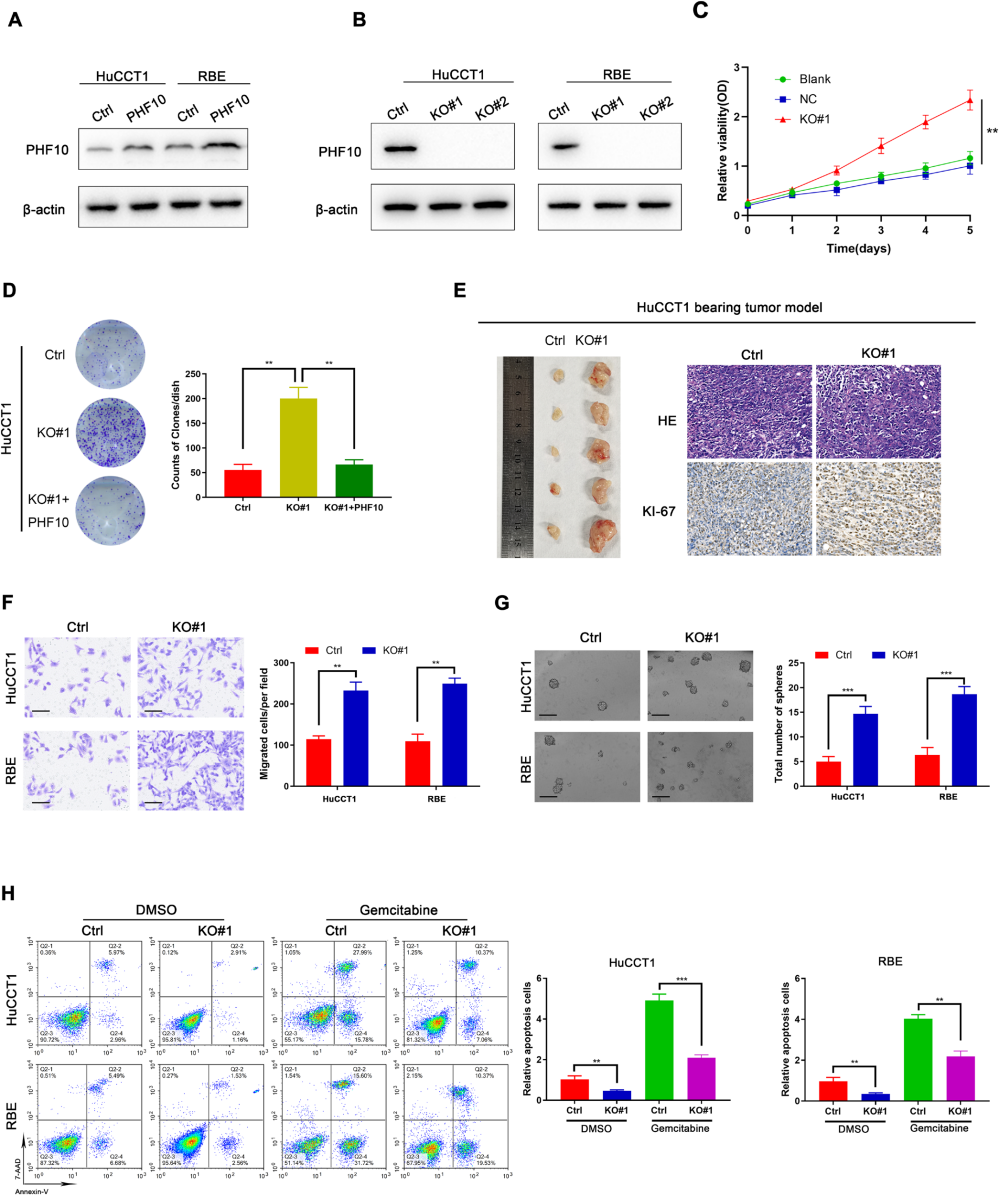
PHF10 deficiency enhanced cell proliferation, migration and chemotherapy resistance of CHOL. (A) The protein level of PHF10 in HuCCT1 and RBE cells with PHF10 overexpression were determined by western blotting. (B) After PHF10 knockout by sgRNAs in two CHOL cell lines, PHF10 expression was detected using western blotting assay. (C) The CCK‐8 assay was conducted to detect the cell viability of cells transfected with vector or PHF10. (D) Clone formation assay was conducted to determine cell proliferation of PHF10‐deleted cells transfected with vector or PHF10, respectively. (E) Representative tumour pictures of PHF10‐KO and control nude mice (*N* = 5/group). The HE pictures and Ki‐67 staining were listed on the right. (F) Transwell assay was conducted to detect the cell migration ability of cells with WT or PHF10 deficiency, respectively. Scale bar = 100 μm. (G) Sphere formation assay was conducted to confirm the self‐renewal ability of cells with WT or PHF10 deficiency, respectively. Scale bar = 500 μm. (H) Cell apoptosis was detected by Annexin V/7‐AAD double staining. Quantification of cell apoptosis rates was exhibited on the right. *p < 0.05, **p < 0.01, ***p < 0.001.

To explore the regulatory role of PHF10 on CHOL cells, we first established GFP‐tagged PHF10‐overexpressing HuCCT1 and RBE cells via lentivirus infection technology (Figure 2A). Besides, we also transduced HuCCT1 and RBE cells with two distinct sgRNAs targeting PHF10, which were confirmed by western blot (Figure 2B). Knock‐out of PHF10 significantly promoted cell growth, as indicated by CCK‐8 assays (Figure 2C and Figure S2A). The colony formation abilities of CHOL cells were also significantly enhanced with PHF10 knock‐out compared with control cells (Figure 2D and Figure S2B). In line with the in vitro findings, we also constructed the HuCCT1 tumour bearing model and found that PHF10 deficiency remarkably promoted tumour growth in the tumour xenograft studies relative to tumours derived from control cells, as reflected by tumour Ki‐67 staining expressions and tumour volumes (Figure 2E). Furthermore, the wound healing experiments suggested that PHF10 inhibition enhanced CHOL cell migration ability (Figure S2C), and transwell assays also indicated that PHF10 deficiency reinforced CHOL cell invasion (Figure 2F). Compared with control cell, PHF10‐deleted HuCCT1 and RBE cells further exhibited enhanced cancer stem cell (CSC)‐like properties, like self‐renewal ability (Figure 2G). Given that tumour stemness features may contribute to chemotherapy tolerance across multiple malignancies as reported, we thus treated the cells with Gemcitabine, which constitutes the baseline chemotherapy treatments for CHOL patients. As indicated by the results from flow cytometry, we found that knockout of PHF10 significantly attenuated cell apoptosis in CHOL cells (HuCCT1 and RBE) treated with either DMSO or Gemcitabine (Figure 2H). Collectively, our data supported that PHF10 may be a tumour suppressor and PHF10 deficiency may notably enhance tumour proliferation, migration, self‐renewal ability, as well as chemotherapy resistance.

### 3.3 PHF10 Coordinates With SETDB1 to Restrict HMGB1 Levels via H3K9me3 Modifications

**FIGURE 3:**
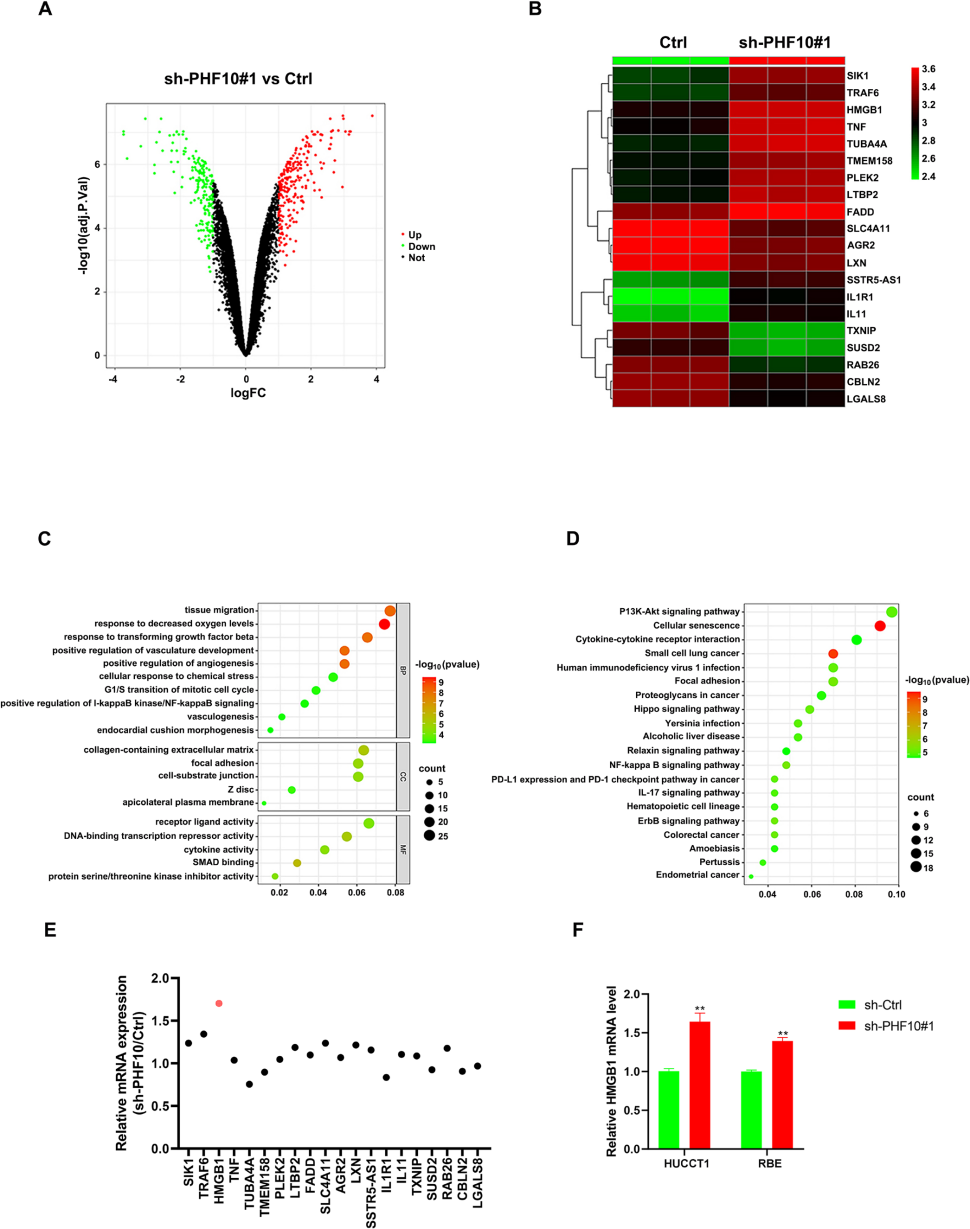
High‐throughput sequencing data indicated low PHF10 activated multiple oncogenic crosstalk, including NF‐κB signalling. (A) Volcano plot exhibiting the differentially expressed genes (DEGs) between control and sh‐PHF10#1 HuCCT1 cells. (B) Heatmap plot showing the cluster analysis of altered DEGs. (C) Gene ontology (GO) analysis indicated the enriched crosstalk based on the DEGs. (D) KEGG analysis indicated the enriched crosstalk based on the DEGs. (E) RT‐qPCR assay validated the altered mRNA level of 20 DEGs in control and sh‐PHF10#1 cells. (F) Validations of HMGB1 mRNA level in another two sh‐PHF10 cells via qPCR technology. *p < 0.05, **p < 0.01, ***p < 0.001.

To gain further insights into the underlying mechanisms, we performed the transcriptome profiling analysis in PHF10‐depleted HuCCT1 cells and control cells. Differential analysis was conducted to found a list of 434 altered genes in PHF10‐deficient cells with |Log2(Foldchange)| > 1 and *p* value < 0.01. The volcano plot futher illustrated the distributions of differentially expressed genes (DEGs), where up‐regulated genes were noted in red and down‐regulated genes were noted in green (Figure 3A). The heatmap plot further revealed the top 20 DEGs with the smallest adjusted *p*‐values between control and sh‐PHF10#1 groups (Figure 3B). In addition, the DEGs were used to perform the Gene ontology (GO) analysis, in which G1/S transition of mitotic cell cycle, positive regulation of I‐kappaB kinase/NF‐kappaB signalling, focal adhesion, and DNA‐binding transcription repressor activity were significantly enriched (Figure 3C). At the same time, differential genes were also enriched by KEGG pathway analysis, and the results showed that differential genes were enriched in pathways including NF‐kappaB signalling pathway (Figure 3D). We also detected 20 DEGs and observed that HMGB1 mRNA expressions had the sharpest increase upon PHF10 deficiency, as revealed by the RT‐qPCR assays (Figure 3E). Meanwhile, we observed the elevated HMGB1 mRNA levels in HuCCT1 and RBE cells with PHF10 knockdown (Figure 3F). Considering that HMGB1 was a well‐known regulator of NF‐κB signalling, we thus focused on the regulations between PHF10 and HMGB1 downstream targets.

**FIGURE 4:**
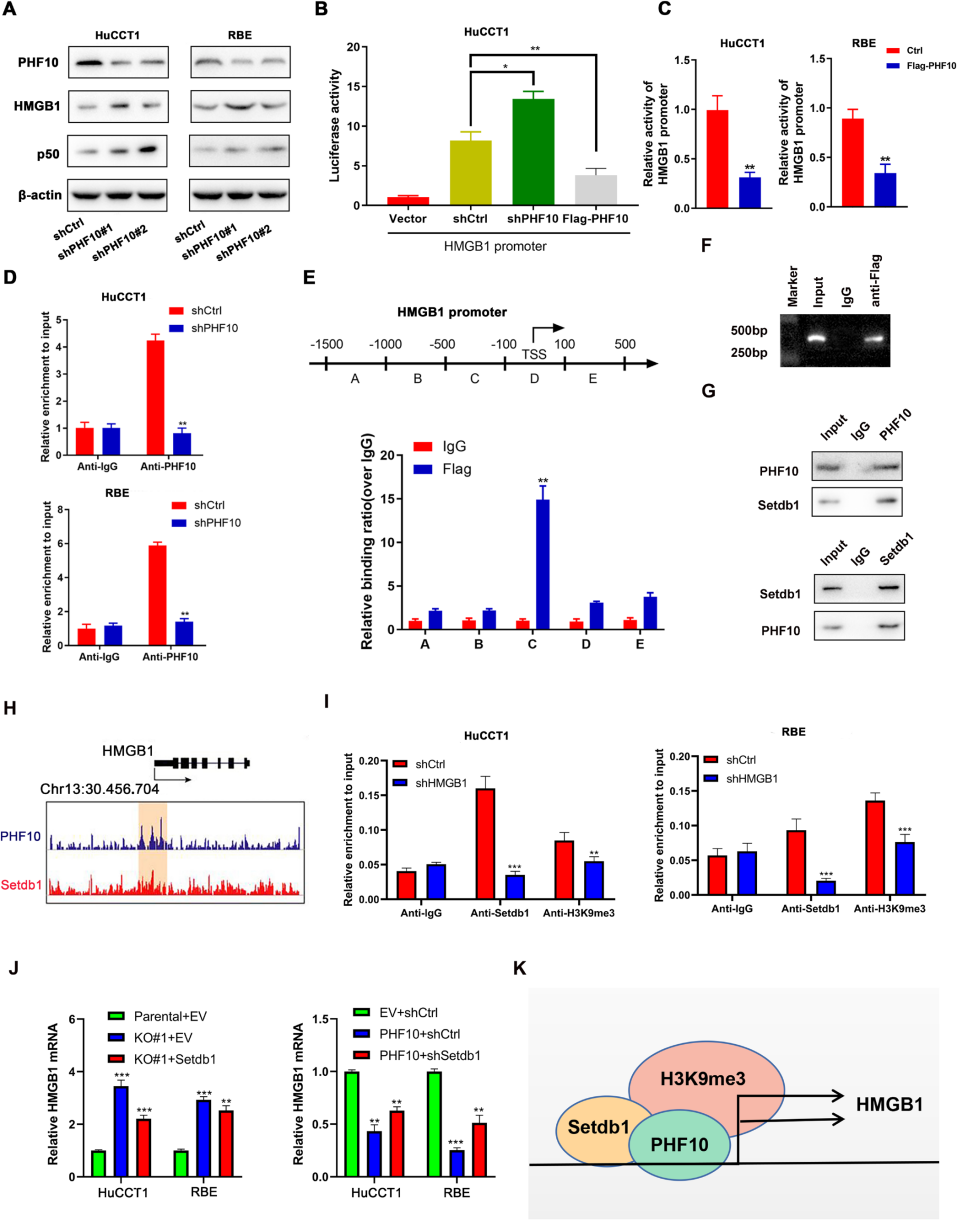
PHF10 restricts HMGB1 transcriptional level via directly binding to its promoter region. (A) Detection of HMGB1 and p50 protein level in shCtrl and shPHF10 cells via western blotting. (B) The HMGB1 promoter sequences were cloned into the pGL3 plasmid and co‐transfected with shPHF10/Flag‐PHF10 plasmids. After 2 days transfection, luciferase activity was detected, where the pGL3‐basic vector was utilised as the control. (C) Dual‐luciferase reporter assay was performed to determine the HMGB1 promoter activity upon PHF10 overexpression in HuCCT1 and RBE cells. (D) ChIP‐qPCR assay were conducted to detect the occupancy of PHF10 and the HMGB1 promoter in HuCCT1 and RBE cells, where IgG was used as a negative control. (E, F) ChIP assay was conducted using the Flag antibody to identify specific PHF10‐binding sites within HMGB1 promoter region, where IgG was used to be the negative control. (G) Western blot of indicated proteins in WCL and Co‐IP samples of IgG or anti‐PHF10 antibody obtained from the cell extracts of HuCCT1 cells treated with 20 μM of MG132 for 8 h (upper panel). The converse Co‐IP using anti‐Setdb1 antibody was conducted on the lower panel. (H) The co‐occupy of Setdb1 and H3K9me3 modifications was illustrated on the HMGB1 promoter via the Cistrome DB resource. (I) ChIP‐qPCR assays were conducted to detect the occupancy of Setdb1 and H3K9me3 modification at the HMGB1 promoter in HuCCT1 and RBE cells, where IgG was used as a negative control. (J) The RT‐qPCR assay was conducted to detect the HMGB1 mRNA level in HuCCT1 and RBE cells, including three groups like parental+EV, KO#1 + EV and KO#1 + Setdb1. (K) The illustration of the model showing PHF10/Setdb1 axis in regulating HMGB1 levels in CHOL cells. *p < 0.05, **p < 0.01, ***p < 0.001.

In consistent with the above findings, we found cells with stable PHF10 knockdown exhibited notably increased expressions of HMGB1 and p50 via western blot (Figure 4A). Thus, the dual luciferase reporter experiment was conducted to determine the regulations by which PHF10 modulates HMGB1 expression. Then, the fragments of HMGB1 promoter regions were cloned into the pGL3‐basic vector. Afterwards, the shPHF10/Flag‐PHF10 plasmids and the HMGB1 promoter were co‐transfected into HuCCT1 cells, where the empty pGL3‐basic vector was considered to be the control. As expected, HMGB1 promoter activity was remarkably elevated via PHF10 knockdown, which was also notably reduced when ectopic Flag‐PHF10 was co‐transfected (Figure 4B). We further demonstrated that PHF10 overexpression could suppress the activity of HMGB1 promoter in HuCCT1 and RBE (Figure 4C). Furthermore, Chromatin immunoprecipitation (ChIP) assay using the specific antibodies of PHF10 and H3K9me3 was further carried out to find that PHF10 deficiency could significantly suppress the enrichment of H3K9me3 on HMGB1 promoter (Figure 4D). As indicated in Figure 4E,F, we further identified that PHF10‐binding loci were mainly enriched in region C (−500 to −100 bp) of the HMGB1 promoter. Collectively, these results showed that PHF10 suppressed HMGB1 transcription via directly binding to its proximal promoter region, co‐operating with H3K9me3 enrichment. As is well documented, Setdb1 is a protein lysine methyltransferase and methylates histone H3 at lysine 9 (H3K9). Setdb1 and Setdb1‐mediated H3K9 trimethylation (H3K9me3) exert essential roles for silencing of endogenous and exogenous retroelements, thus contributing to genome stability against retroelement transposition. We thus speculated that whether Setdb1 participated in the PHF10‐mediated H3K9me3 modifications on HMGB1 promoter region. Firstly, the Co‐Immunoprecipitation (Co‐IP) assay was conducted to confirm the endogenous interactions between PHF10 and Setdb1 proteins in HuCCT1 cells (Figure 4G). Besides, we queried the transcriptome data via the Cistrome Data Browser (DB) dataset (http://cistrome.org/db/#/) to observe the co‐occurrence of Setdb1 and H3K9me3 enrichment on the HMGB1 promoter region in Hela cells (Figure 4H). In addition, we conducted the Chromatin immunoprecipitation (ChIP) assay using the specific antibodies of Setdb1 and H3K9me3 to enrich the DNA fragments combined with them and designing the specific primer of HMGB1 promoter to detect the enrichment of HMGB1. We found that the HMGB1 promoter sequences enriched by the two antibodies were significantly reduced in the shSetdb1 group compared with the shCtrl group in HuCCT1 and RBE cells (Figure 4I). Last of all, we found that PHF10 deficiency could elevate HMGB1 mRNA levels, while ectopic expression of Setdb1 suppressed the levels of HMGB1 (Figure 4J). In contrast, PHF10 overexpression inhibited the mRNA levels of HMGB1, which could be rescued by Setdb1 knockdown (Figure 4J). Collectively, these findings suggested that PHF10 recruited Setdb1 to mediate H3K9me3 modifications on the HMGB1 promoter. PHF10 co‐operated with Setdb1 to suppress HMGB1 mRNA levels in CHOL cells (Figure 4K).

### 3.4 Down‐Regulated PHF10 Regulates HMGB1 Levels and Relied on HMGB1 to Drive CHOL Progression

**FIGURE 5:**
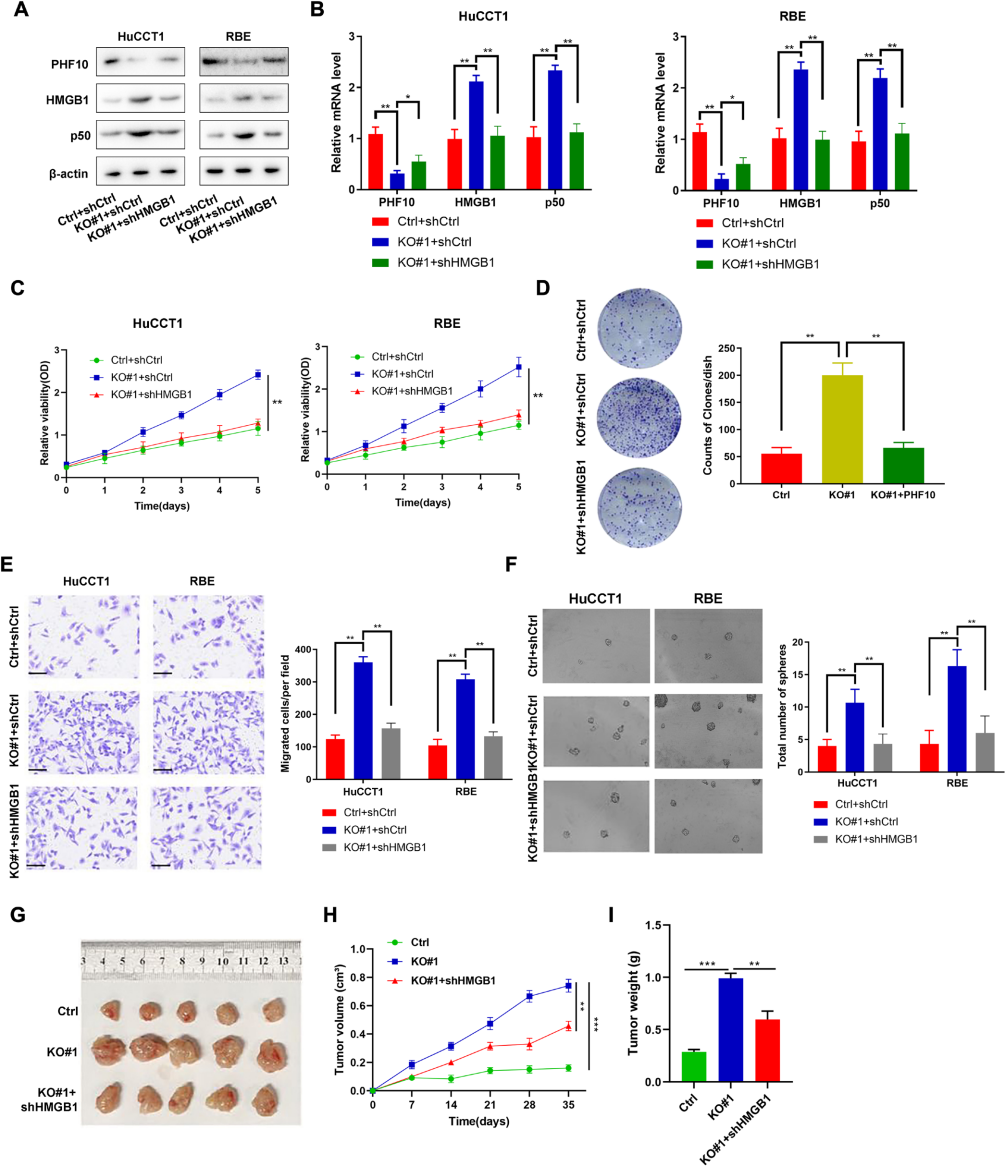
PHF10 deficiency activated HMGB1/p50 axis and depended on HMGB1 to drive CHOL progression. (A) Western blot assays were conducted to detect the HMGB1/p50 levels in PHF10‐deficient cells transfected with shCtrl and shHMGB1. (B) qPCR assays were conducted to confirm the p50 mRNA levels in in PHF10‐deficient cells transfected with shCtrl and shHMGB1. (C) CCK‐8 assays were conducted to monitor the proliferation rates of PHF10‐KO cells transfected with shCtrl and shHMGB1. (D–F) The colony formation ability, migration ability, as well as self‐renewal ability were detected in PHF10‐KO cells, which were transfected with shCtrl and shHMGB1, respectively. (G) The representative graph showed the in vivo subcutaneous tumour model in three indicated groups. (H) The quantification of volumes showed the in vivo growth of tumours derived from three groups. (I) The quantification of tumour weight in three indicated groups. *p < 0.05, **p < 0.01, ***p < 0.001.

To further figure out the potential associations between PHF10 and HMGB1/NF‐κB axis, we detected that p50 levels were notably increased in PHF10‐deleted CHOL cells, which could be successfully restored with HMGB1 inhibition (Figure 5A). In line with this finding, we also observed the same results in Figure 5B, suggesting that PHF10 deficiency could activate p50 expressions and downstream signalling that depends on HMGB1. Besides, we also inhibited HMGB1 in PHF10‐deficient cells to perform the CCK‐8 assays, in which HMGB1 knockdown could remarkably suppress the increased cell growth in PHF10‐deleted CHOL cells (Figure 5C). In addition, in consistent with the results, the colony formation ability, migration ability, as well as self‐renewal ability were enhanced with PHF10 depletion, whereas HMGB1 knockdown impaired these tumour‐promoting properities (Figure 5D–F). Last of all, we also performed the subcutaneous tumour model and found that targeting HMGB1 could suppress the in vivo growth of tumours derived from the PHF10‐deleted cells, as evidenced by the quantification of tumour growth curve and tumour weight (Figure 5G–I). Taken together, these data suggested that PHF10 deficiency depended on HMGB1 to maintain NF‐κB signalling and drive CHOL malignant characteristics.

### 3.5 EZH2 Mediated the Epigenetic Silencing of PHF10 in CHOL

**FIGURE 6:**
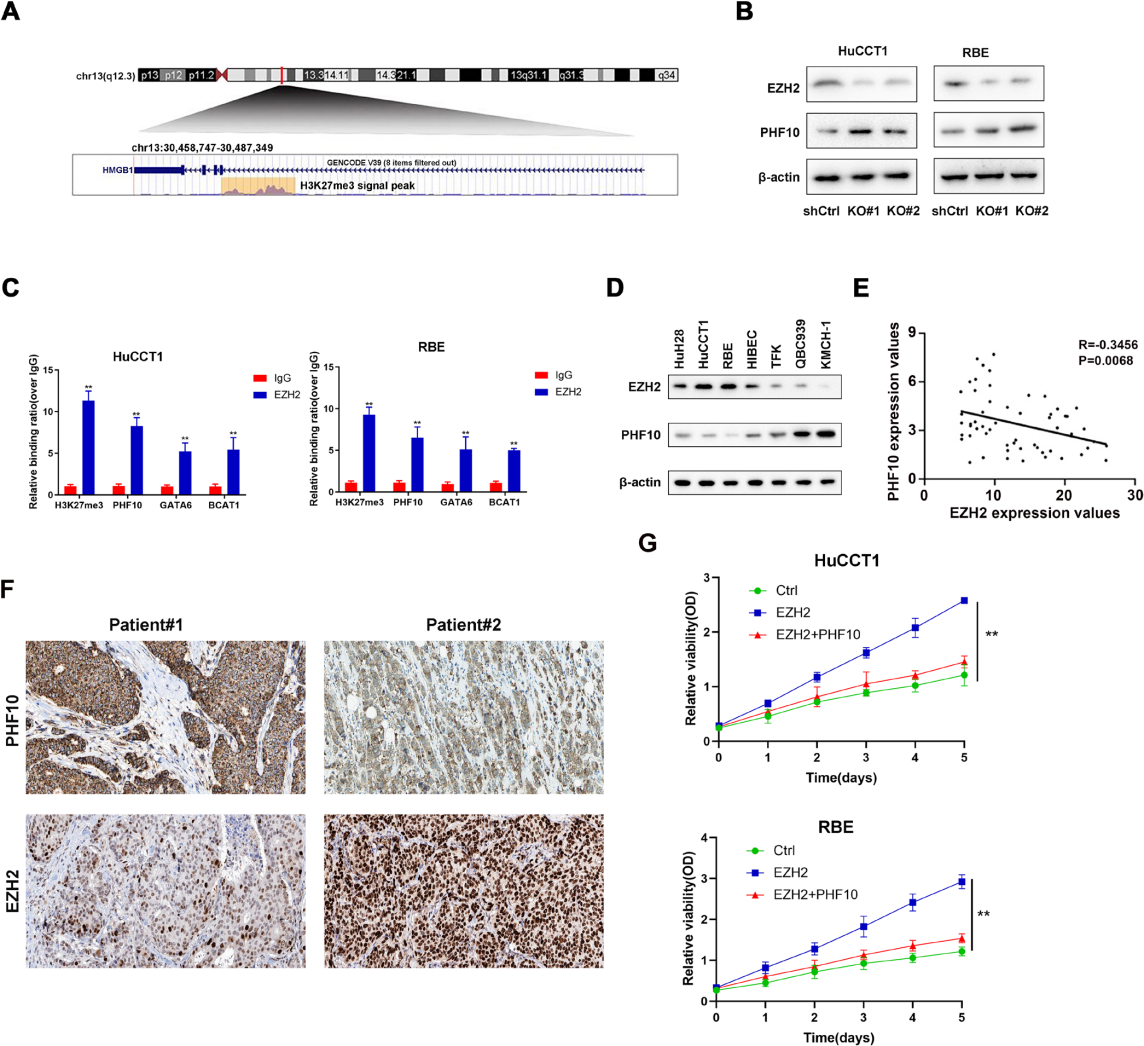
Aberrant EZH2 mediated the epigenetic silencing of PHF10 in CHOL. (A) The enrichment of H3K27me3 in the promoter region of HMGB1 via the UCSC genome bioinformatics site (http://genome.ucsc.edu/). (B) Western blotting assays showing the PHF10 protein levels in EZH2‐knockdown cells. (C) ChIP‐qPCR assays were conducted to confirm the EZH2‐binding at PHF10 promoter regions. (D) Western blotting assay was used to assess PHF10 protein levels in EZH2^high^ and EZH2^low^ CHOL cell lines. (E) Correlation analysis showing the negative associations between EZH2 and PHF10 in 57 clinical tissue specimens. (F) Representative images of negative associations between EZH2 and PHF10 via IHC based on collected samples. (G) CCK‐8 assays suggested the cell proliferation rates in indicated groups. *p < 0.05, **p < 0.01, ***p < 0.001.

To elucidate the in‐depth reasons that contribute to low PHF10 expression in CHOL, we first focused on the modification in the promoter of PHF10 by the querying the UCSC genome dataset (http://genome.ucsc.edu/) (Figure 6A). Given that EZH2 expresses highly in CHOL and is a mast regulator that catalyses H3‐Lys27 methylation to inhibit multiple downstream genes, we thus detected that PHF10 expressions notably increased in EZH2‐knockdown cells (Figure 6B). Besides, ChIP‐qPCR assays were conducted to confirm that EZH2 markedly increased H3K27me3 levels at the PHF10 and other known representative EZH2‐targets, like GATA6 and CHOLT1 (Figure 6C). Moreover, negative associations between EZH2 and PHF10 expression levels were further confirmed in CHOL cell lines (Figure 6D) and 57 clinical tissue specimens (Figure 6E). Immunohistochemical results of representative clinical samples were presented (Figure 6F). Accordingly, we also found that overexpression of PHF10 could markedly impair the cell proliferation ability of EZH2‐overexpressing cells (Figure 6G). Taken together, our results indicated that EZH2 restricts the PHF10 expressions in CHOL and PHF10 suppression may partly account for the malignant features of EZH2^high^ CHOL.

### 3.6 Targeting HMGB1 (Glycyrrhizin) Rendered PHF10low CHOL Sensitive To Chemotherapy

**FIGURE 7:**
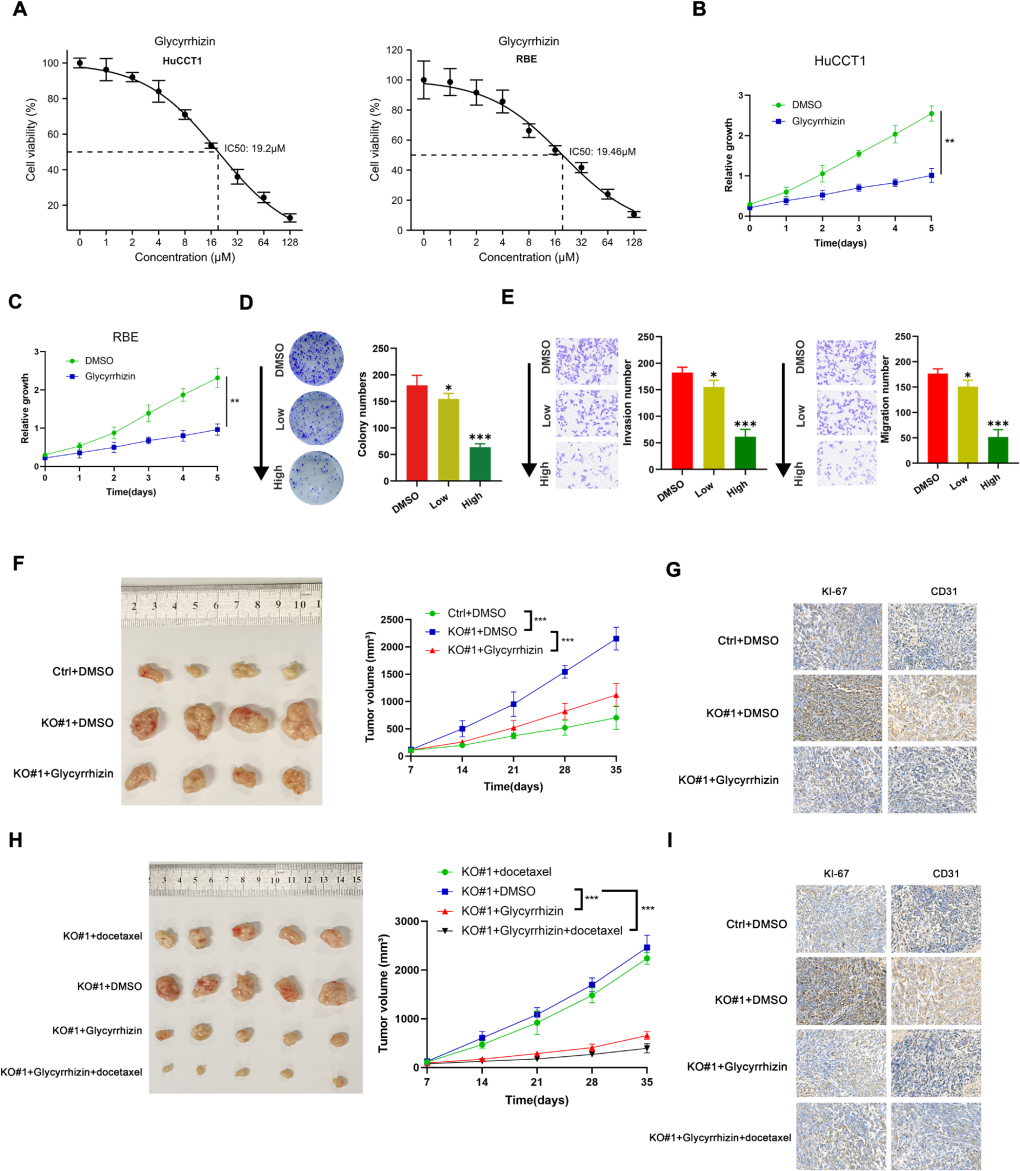
Glycyrrhizin (HMGB1 inhibitor) rendered PHF10^low/−^ CHOL sensitive to chemotherapy. (A) The half maximal inhibitory concentration (IC50) of Glycyrrhizin was confirmed in CHOL cell lines. (B, C) The cell growth rates were detected and compared in cells treated with DMSO and Glycyrrhizin, respectively. (D, E) The colony formation ability and migration ability of CHOL cells were detected under different concentrations of Glycyrrhizin. (F, G) Representative tumour graphs were showed in three indicated groups (F, left). Tumour volumes (F, middle) and Ki‐67 staining levels (G). (H, I) IHMGB1 inhibitor (Glycyrrhizin) was effective to suppress PHF10^low/−^ CHOL in vivo growth and is synergistic with gemcitabine treatment. *p < 0.05, **p < 0.01, ***p < 0.001.

As previously reported, activation of either HMGB1 or NF‐κB crosstalk could lead to multiple drug resistance in tumours, especially chemotherapy. We thus considered to utilise HMGB1 inhibitor (Glycyrrhizin) to suppress HMGB1 and its downstream NF‐κB signalling. We firstly determined the half maximal inhibitory concentration (IC50) of Glycyrrhizin in CHOL cell lines (Figure 7A). Similar to shRNAs targeting HMGB1, the selective HMGB1 inhibitor (Glycyrrhizin) decreased proliferation rate of PHF10‐deleted cells (Figure 7B,C). Intriguingly, Glycyrrhizin could notably suppress the clone formation ability or migration ability of PHF10‐deleted cells in a dose‐dependent manner when cells were treated with 5 μM(Low) or 20 μM(High) Glycyrrhizin (Figure 7D,E). In line with in vitro findings, Glycyrrhizin(20 μM) was effective to inhibit the growth of in vivo tumours derived from PHF10‐deleted CHOL cells, as reflected by tumour volumes, Ki‐67 and CD31 staining levels (Figure 7F,G). Lastly, we further treated the mice with Glycyrrhizin or gemcitabine to assess the sensitivity. As shown in Figure 7H,I, different from Glycyrrhizin, gemcitabine exerted limited effects on tumours derived from PHF10‐deleted CHOL cells, in line with the above results that PHF10 deficiency may contribute to chemotherapy tolerance. Notably, Glycyrrhizin treatment (10 mg/Kg body weight) could render PHF10‐deleted cells sensitive to Gemcitabine, suggesting that Glycyrrhizin has synergistic efficacy with Gemcitabine. In conclusion, we found that HMGB1 inhibitor (Glycyrrhizin) was specifically suitable for treating PHF10^low/−^ CHOL and could further enhance gemcitabine sensitivity.

## 4 Discussion

Cancer belongs to a genetic and epigenetic disease. Epigenetic mechanisms modulate various processes of cancer biology, from promoting primary tumour growth and invasion to modulating the immune response within the tumour microenvironment [18, 19]. Different from the genetic mutations, which are difficult to interfere, dysregulated epigenetic mechanisms can be effectively targeted by small molecule compounds [20, 21]. In addition, modulation of the epigenome in multiple solid tumours exposes cancer cells to chromatin impairment, increasing their sensitivity to immunotherapy or chemotherapy [22, 23]. These advantages appealed a growing attention in recent years to developing epigenetic strategies to combat cancer. As reported, the process of a benign and nonmalignant ductal carcinoma in situ to the advanced and metastatic CHOL requires many dysregulated oncogenes and tumour suppressors, each of which endows a unique growth advantage to tumour cells and controls a specific phenotypic characteristic of tumour progression and metastasis [24]. Epigenetic reprogramming plays an essential role in dysregulation of these genes and represents an important mechanism of CHOL progression and metastasis [25, 26]. As a result, it is of great significance to identify essential epigenetic vulnerabilities for CHOL treatment.

In the present study, we found that PHF10 was down‐regulated in CHOL samples based on bioinformatic analysis and low PHF10 predicted inferior survival outcomes of patients. We also validated our findings in collected CHOL samples, in which PHF10 may be an independent prognostic factor. We also demonstrated that PHF10 could notably suppress CHOL proliferation, whereas PHF10 deficiency enhanced malignant features of CHOL cells, like cell proliferation, migration, invasion abilities, as well as self‐renewal capacities. The subcutaneous CHOL tumour models also indicated that PHF10 ablation could potentiate in vivo tumour growth of CHOL cells. Of note, PHF10 deficiency could attenuate notable apoptosis of CHOL cells and render CHOL cells more resistant to gemcitabine treatment. The transcriptome analysis implicated that PHF10 loss mainly altered a series of oncogenic crosstalk and elevated the expressions of top downstream hit HMGB1. The ChIP‐qPCR and Co‐IP assays mainly suggested that PHF10 could recruit and interact with Setdb1 to promote the enrichment of H3K9me3 at the promoter of HMGB1 to restrict its mRNA expressions. PHF10 loss thus impaired the Setdb1‐mediated suppression of HMGB1 and promoted HMGB1 transcriptions via defective H3K9me3 modifications. We further uncovered that down‐regulated PHF10 could also activate NF‐kB signlaing via HMGB1 and relied on HMGB1 to promote CHOL progression. In addition, we further delineated an epigenetic mechanism by which EZH2 could restrict the PHF10 expressions in CHOL and PHF10 suppression may partially contribute to the malignant features of EZH2^high^ CHOL. Last of all, we used HMGB1 inhibitor (Glycyrrhizin) to suppress HMGB1 and its downstream NF‐κB signalling. Previous research has confirmed that glycyrrhizin ameliorates colorectal cancer progression by regulating NHEJ pathway through inhibiting HMGB1‐induced DNA damage response and glycyrrhizin effectively reverses the cancer‐promoting effects of IGF2BP3 overexpression in bladder cancer [27, 28]. We observed that Glycyrrhizin could notably suppress the colony formation ability or migration ability of PHF10‐deleted cells in vitro and in vivo. Importantly, targeting HMGB1 with Glycyrrhizin could render PHF10^low/−^ CHOL cells more sensitive to chemotherapy and possessed synergistic effect when they were combined together.

As the first‐line basic drug, gemcitabine could induce the apoptosis of CHOL cells by distrubing the mitotic process and hindering the DNA replication of cells [29, 30]. It has been commonly used with notable effects in advanced CHOL, pancreatic cancer, bladder cancer and other malignancies [31, 32]. However, multiple studies have indicated that a proportion of CHOL patients would suffer from gemcitabine resistance and the overall efficacy of chemotherapy remains to be limited [33].

High mobility group box 1 (HMGB1) is a highly conservative nucleoprotein and belongs to the group of non‐histone chromatin‐associated protein [34]. HMGB1 exerts essential roles in regulating DNA replication process, transcription, chromatin remodelling when it locates in the nucleus [35]. Cytoplasmic HMGB1 mainly participates in immune responses by increasing autophagy, inhibiting apoptosis, and regulating mitochondrial function [36]. Previous studies have revealed the clinical significance of HMGB1 in CHOL treatment.

Nattawan Suwannakul et al. revealed that the overexpression of subcellular HMGB1 could be associated with the metastatic status of patients with *clonorchis sinensis* CHOL, which was shown to be effective for *clonorchis sinensis* CHOL prognosis [37]. Besides, Yunfei Xu et al. revealed that intracellular HMGB1 could be released from PHCCA cells and promote angiogenesis via elevating VEGFR2 in vessel endothelial cells. The results of the study showed that high expression of HMGB1 was associated with MVD and poor prognosis in clinical analysation andpostoperative serum HMGB1 and cholangitis could predict high recurrence and unfavourable prognosis.

In this study, we found that HMGB1 could activate NF‐kB signalling in CHOL progression, providing a novel mechanism by which HMGB1 enhances CHOL gemcitabine resistance. Intriguingly, targeting HMGB1 could render CHOL cells more sensitive to gemcitabine treatment, suggesting a clinical significance of combination of gemcitabine with HMGB1 inhibitor.

However, this study still has some shortcomings that need to be further improved. First of all, we should collect more CHOL samples in our hospital to assess the optimal cutoff that categorises PHF10^high^ and PHF10^low^ groups. Besides, we are still uncertain about the underlying mechanisms between PHF10 and immune infiltrations of CHOL tumour microenvironment. In addition, more pre‐clinical mice models and patient‐derived organoids were warranted to assess the drug efficacy of Glycyrrhizin for CHOL and the synergistic effect with gemcitabine.

In summary, our study identifies that PHF10 suppressed CHOL development and metastasis via modulating HMGB1/NK‐kB signalling, which is also regulated by EZH2. Down‐regulated PHF10 impaired the setdb1‐mediated suppression of HMGB1, thus triggering the epigenetic remodelling in CHOL. Therefore, our study suggested that the EZH2/PHF10/HMGB1 axis may be used as novel biomarkers and therapeutic vulnerabilities for CHOL treatment.

## Author Contributions

**Qiushi Yin:** conceptualization (equal). **Daning Lin:** methodology (equal), writing – original draft (equal). **Weiqian Zeng:** data curation (equal), methodology (equal). **Shijing Gu:** data curation (equal). **Chuangshi Zhu:** data curation (equal), supervision (equal). **Changfu Liang:** validation (equal), writing – original draft (equal). **Yan Yang:** conceptualization (equal), resources (equal), supervision (equal).

## Acknowlegements

The authors have nothing to report.

## Ethics Statement

Sample collection and usage were approved by the Ethics Committee of the First Affiliated Hospital of Hainan Medical University in accordance with the Declaration of Helsinki (Number: 2018(Keyan) No.2), and written informed consent was obtained from all enrolled patients. All the animal experiments were approved in advance by the Committee for Animal Experimentation of the First Affiliated Hospital of Hainan Medical University (Nubmer: HYLL‐2020‐010).

## Conflicts of Interest

The authors declare no conflicts of interest.

## Supporting information


Figure S1.
Figure S2.

## Data Availability

The data that support the findings of this study are available from the corresponding author upon reasonable request.
