# Supplementary material for: EZH2‐Mediated PHF10 Suppression Amplifies HMGB1/NF‐κB Axis That Confers Chemotherapy Resistance in Cholangiocarcinoma
Source: J Cell Mol Med. 2025 Feb 4;29(3):e70363. doi: 10.1111/jcmm.70363 (PMC11794005; doi:10.1111/jcmm.70363)
Supplement: Supplementary file 1 — Figure S1. Figure S2. [file JCMM-29-e70363-s001.docx]

**
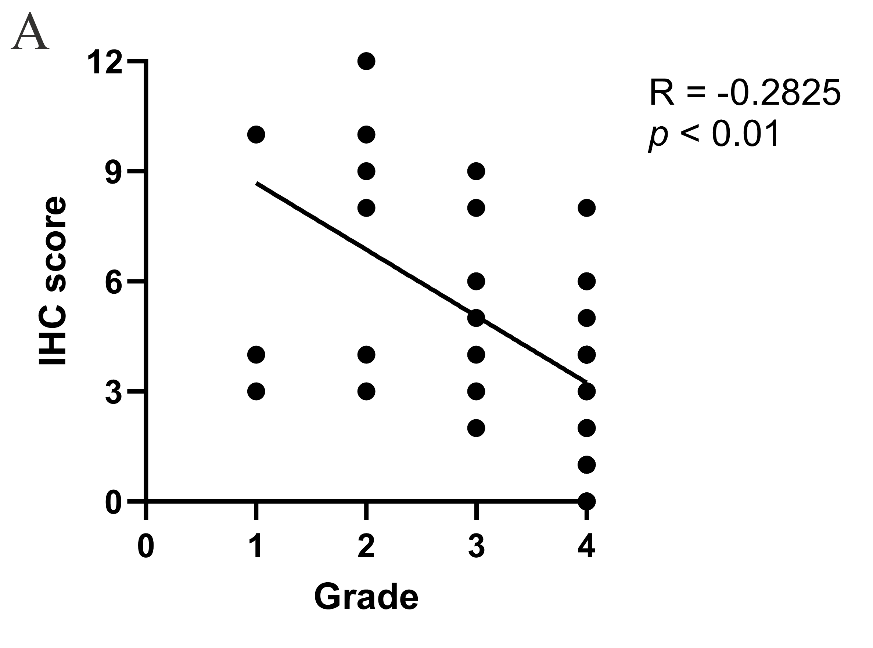
**

**Supplementary Figure 1. Fig. 1 PHF10 was down-regulated in CHOL that predicts poor prognosis.**

A Correlation analysis of tumor grade and immunohistochemical score in 60 patients

**
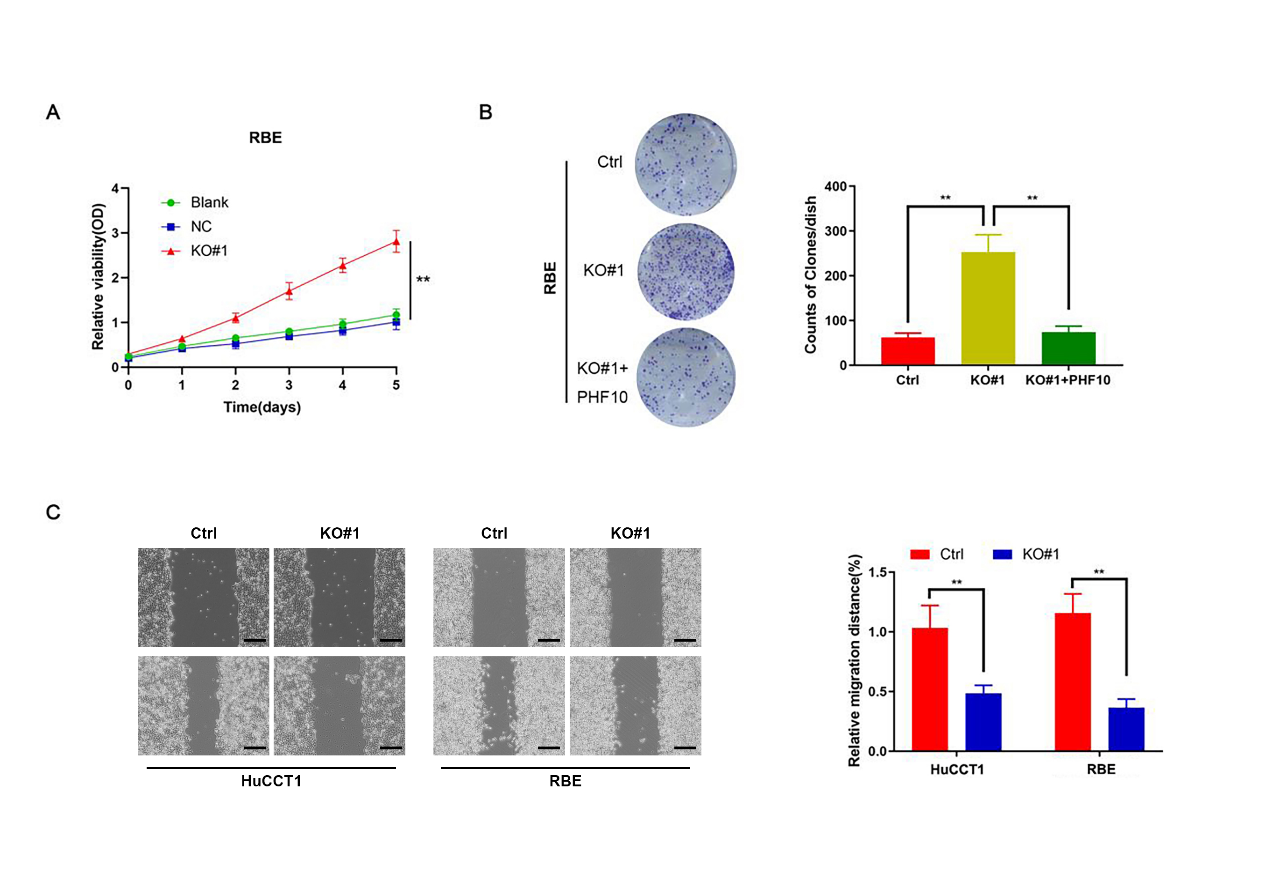
**

**Supplementary Figure 2. Targeting PHF10 enhanced tumor progression.**

**A** CCK-8 assays were conducted to detect cell growth of cells transfected with vector and PHF10. **B** Colony formation assays were conducted to detect cell growth of cells transfected with vector and PHF10. **C** Wound healing assays were conducted in indicated cells. **P* < 0.05, ***P* < 0.01, ****P* < 0.001.
